# Salicylate improves macrophage cholesterol homeostasis via activation of Ampk[S]

**DOI:** 10.1194/jlr.M058875

**Published:** 2015-05

**Authors:** Morgan D. Fullerton, Rebecca J. Ford, Chelsea P. McGregor, Nicholas D. LeBlond, Shayne A. Snider, Stephanie A. Stypa, Emily A. Day, Šárka Lhoták, Jonathan D. Schertzer, Richard C. Austin, Bruce E. Kemp, Gregory R. Steinberg

**Affiliations:** * Divisions of Endocrinology and Metabolism McMaster University, Hamilton, Canada; †† Nephrology, McMaster University, Hamilton, Canada; † Department of Medicine, and Departments of Biochemistry and Biomedical Sciences McMaster University, Hamilton, Canada; §§ Pediatrics, McMaster University, Hamilton, Canada; § Department of Biochemistry, Microbiology, and Immunology, University of Ottawa, Ottawa, Canada; ** Hamilton Centre for Kidney Research, St. Joseph’s Healthcare Hamilton, Hamilton, Canada; *** St. Vincent’s Institute of Medical Research and Department of Medicine, University of Melbourne, Fitzroy, Australia

**Keywords:** cholesterol efflux, adenosine 5′-monophosphate-activated protein kinase, lipid homeostasis, atherosclerosis, reverse cholesterol transport

## Abstract

Atherosclerosis stems from imbalances in lipid metabolism and leads to maladaptive inflammatory responses. The AMP-activated protein kinase (Ampk) is a highly conserved serine/threonine kinase that regulates many aspects of lipid and energy metabolism, although its specific role in controlling macrophage cholesterol homeostasis remains unclear. We sought to address this question by testing the effects of direct Ampk activators in primary bone marrow-derived macrophages from Ampk β1-deficient (β1^−/−^) mice. Macrophages from Ampk β1^−/−^ mice had enhanced lipogenic capacity and diminished cholesterol efflux, although cholesterol uptake was unaffected. Direct activation of Ampk β1 via salicylate (the unacetylated form of aspirin) or A-769662 (a small molecule activator), decreased the synthesis of FAs and sterols in WT but not Ampk β1^−/−^ macrophages. In lipid-laden macrophages, Ampk activation decreased cholesterol content (foam cell formation) and increased cholesterol efflux to HDL and apoA-I, effects that occurred in an Ampk β1-dependent manner. Increased cholesterol efflux was also associated with increased gene expression of the ATP binding cassette transporters, Abcg1 and Abca1. Moreover, in vivo reverse cholesterol transport was suppressed in mice that received Ampk β1^−/−^ macrophages compared with the WT control. Our data highlight the therapeutic potential of targeting macrophage Ampk with new or existing drugs for the possible reduction in foam cell formation during the early stages of atherosclerosis.

Atherosclerosis is a chronic condition that stems from the delivery and unregulated uptake of circulating lipoproteins by macrophages in the vasculature. The retention and subsequent modification (oxidation, acetylation, or aggregation) of LDL particles results in the activation of endothelial cells at the branch points of arteries and increases the expression of various adhesion molecules, as well as chemoattractant proteins (1). This facilitates the recruitment and transmigration of circulating monocytes from the lumen to the subintimal space [for review see (2, 3)]. Infiltrating monocytes differentiate to macrophages (4) and upregulate scavenger receptors (5, 6) [such as scavenger receptor B1 (SR-B1), scavenger receptor-A (SR-A), and scavenger receptor CD36 (CD36)], which are critical for the uptake of modified lipoproteins within the subendothelial space (7). The accumulation of lipid-laden macrophages (foam cells) results in the progression of atherosclerotic plaques. As atherosclerosis progresses, increased lipid content (mainly cholesteryl esters) of macrophages is intimately linked to the increased inflammatory tone in the plaque environment, and the infiltration and activation of numerous innate and adaptive immune cells (2, 3, 5, 8). Because the lipid burden of macrophage foam cells contributes directly to the inflammatory tone and risk of plaque rupture (3), strategies aimed at lowering the lipid content of foam cells may be useful therapeutically.

The physiological process known as reverse cholesterol transport (RCT), whereby cholesterol is removed from peripheral tissues and transported by HDLs to the liver for excretion through bile and ultimately the feces (9), has garnered therapeutic interest. In the macrophage, excess cholesterol is esterified to cholesteryl esters and stored in lipid droplets, leading to foam cell formation. However, cholesterol can be mobilized from lipid droplet stores (10, 11) and effluxed via active transport to extracellular acceptors, HDL and lipid-poor apoA-I, a process mediated by the ATP binding cassette transporters, Abcg1 and Abca1, respectively (12–14). There is now strong evidence to suggest that increased cholesterol efflux from macrophage foam cells is a strong predictor of improved CVD risk profile (9, 15–17). Thus a mechanistic understanding of signaling pathways that promote cholesterol efflux and RCT is crucial for developing future therapeutic interventions.

AMP-activated protein kinase (Ampk) is a highly conserved serine/threonine kinase that activates catalytic processes to generate ATP (such as glucose uptake and FA oxidation), and inhibits anabolic pathways (such as lipid and protein synthesis) [for review see (18, 19)]. In mammals, Ampk exists as an αβγ heterotrimer (20), where the β subunit acts as a scaffold for the α-catalytic and γ-regulatory subunits (21). While there are many therapeutics [including the anti-diabetic drug metformin (22, 23)] that activate Ampk indirectly, the small molecule, A-769662 (24), and salicylate (25) increase Ampk activity directly through the β1 subunit drug binding site (25–27). The activation of Ampk results in the inhibitory phosphorylation of acetyl-CoA carboxylase (Acc) (28, 29) and HMG-CoA reductase (Hmgcr) (30, 31), the rate-limiting enzymes controlling FA and cholesterol biosynthesis, respectively. Macrophages exclusively express an Ampk α1β1γ1 heterotrimer, which is essential for controlling rates of FA oxidation and reducing inflammation (32, 33); however, the importance of Ampk in controlling cholesterol metabolism has not been studied.

Aspirin (acetylsalicylate) is one of the most widely prescribed medications world-wide for the primary and secondary treatment of CVD. Aspirin irreversibly inhibits cyclo-oxygenases to disrupt prostaglandin synthesis (34) and reduces coagulation by inhibiting thromboxane A_2_ production in platelets (35). It was initially thought that the benefits to aspirin therapy were entirely mediated by its anti-platelet effects; however, genetic disruption of thromboxane A_2_ synthesis and other anti-coagulant therapies do not display the same cardioprotective effect (36, 37). Upon ingestion, aspirin is rapidly deacetylated in the circulation to salicylate (38). Given the gastrointestinal and thrombotic side-effects of higher doses of aspirin and salicylate, a pro-drug of salicylate (salsalate), which is better tolerated, is now being used in clinical trials for CVD (TINSAL-CVD: NCT00624923). At clinical salsalate concentrations, salicylate directly activates Ampk and is required for increasing rates of FA oxidation in hepatocytes (25). The role of salicylates in regulating macrophage Ampk activity has not been studied.

Here we show that deletion of Ampk results in higher FA and cholesterol synthesis. Under lipid-laden conditions, Ampk β1-deficient (β1^−/−^) macrophages have more lipid accumulation and lower cholesterol efflux. In addition, direct activation of Ampk was able to restore cholesterol homeostasis in lipid-loaded macrophages through the suppression of lipid synthesis and foam cell formation, as well as stimulation of cholesterol efflux in vitro and in vivo.

## MATERIALS AND METHODS

### Mice

The generation and characterization of the Ampk β1^−/−^ mice has been previously described (39, 40). The Ampk β1^−/−^ and littermate WT control mice used in these studies were housed in specific pathogen-free micro-isolators, maintained on a 12 h light/12 h dark cycle with lights on at 0700, and had unlimited access to standard rodent chow and water. All animal experimental protocols used were approved by the McMaster University Animal Ethics Research Board and the University of Ottawa Animal Care Committee.

### Cell culture

Bone marrow-derived macrophages (BMDMs) were generated as previously described (32). Briefly, mice were euthanized, tibias and femurs isolated, and the ends of each bone cut off. The tibia and femur from each leg were placed into a sterile 0.5 ml microfuge tube that had a hole punctured in the end with an 18 gauge needle, which was then placed inside a 1.5 ml microfuge tube before the addition of 100 μl of DMEM (Invitrogen) to the 0.5 ml tube. Bone marrow cells were collected by centrifuging at 2,000 rpm for 4 min, resuspended and plated in 100 ml of DMEM supplemented with 10% FBS (Invitrogen) and 1% penicillin/streptomycin (Invitrogen) in a T175 flask, and incubated at 37°C in a humidified atmosphere at 5% CO_2_. After 4 h, cells were plated into 10 cm tissue culture dishes in the presence of 20% L929 medium (as a source of macrophage colony stimulating factor) and left to differentiate for 7–8 days. One day prior to the experiment, cells were lifted into suspension in the existing L929-supplemented DMEM by gentle scraping and seeded into the appropriate plate for subsequent experiments.

### Foam cell formation and lipid determinations

BMDMs were subjected to two distinct foam cell protocols. In foam cell protocol 1, BMDMs were incubated in the presence or absence of acetylated LDL (acLDL) (50 μg/ml) [Biomedical Technologies Inc. (BTI)] for 30 h in the presence or absence of salicylate (3 mM) or A-769662 (100 μM). In foam cell protocol 2, cells were incubated with acLDL (50 μg/ml) for 30 h and then equilibrated for 12–16 h in 0.2% BSA DMEM stimulated in the presence or absence of salicylate (3 mM) and A-769662 (100 μM). At the completion of the incubation, cells were washed twice with PBS and cholesterol determined using the Amplex cholesterol kit (Invitrogen) as described (41).

### Cholesterol efflux experiments

acLDL (50 μg/ml; BTI) was pre-equilibrated with 5 μCi/ml [^3^H]cholesterol (Perkin Elmer) in DMEM supplemented with 5% lipoprotein deficient serum (LPDS) (BTI) for 12 h. BMDMs were then incubated with this radioactive mixture for a further 30 h. Radioactive medium was then removed and washed twice with PBS. Cells were then equilibrated in DMEM-LPDS supplemented with 0.2% BSA overnight (16 h) in the presence or absence of salicylate (3 mM), A-769662 (100 μM), or DMSO vehicle control. Fresh medium was then replenished in the presence or absence of 0.2% BSA, recombinant human apoA-I (5 μg/ml- BTI), or human HDL (50 μg/ml; BTI), in the presence or absence of Ampk activators. After 24 h, medium was removed and radioactivity determined by liquid scintillation counting (LSC). Cells were lysed with 0.1 M NaOH and radioactivity determined by LSC. Efflux is expressed as a percentage of dpm for [^3^H]cholesterol in medium/([^3^H]cholesterol in medium + [^3^H]cholesterol in cells) × 100%. Efflux to either apoA-I or HDL was calculated by subtracting the effluxes of the wells containing only BSA without apoA-I or HDL from those containing it. The specific activity for each condition (genotype and treatment) was determined by assessing cholesterol mass and radioactivity prior to the addition of the apoA-I and HDL.

### acLDL uptake

acLDL uptake was measured by determining the uptake of fluorescence-labeled acLDL (Dil-acLDL; BTI) as described previously (42). Briefly, BMDMs were plated into 24-well dishes and foam cells were induced by incubation with acLDL (50 μg/ml; BTI) for 30 h in the presence or absence of salicylate (3 mM), A-769662 (100 μM), or DMSO vehicle control. After foam cell formation, lipid-containing medium was removed and cells were incubated with Dil-acLDL (10 μg/ml) for 2 h. Dil-fluorescence was extracted using isopropanol and relative fluorescence intensity determined at 524 nm (excitation) and at 567 nm (emission) (43).

### Lipogenesis and lipid extraction

BMDMs were labeled with [^3^H]sodium acetate in the presence of 5 mM unlabeled sodium acetate, in the presence or absence of salicylate (3 mM), A-769662 (100 μM), or DMSO vehicle control for 16 h. Cells were then washed twice with ice-cold PBS and scraped in 200 μl PBS for lipid extraction. Lipids were extracted as described previously (44, 45). Radioactivity was determined by counting the chloroform phase containing total lipids or after saponification and extraction with petroleum ether, as previously described (45).

### RNA isolation, cDNA synthesis, and quantitative PCR

RNA was isolated using TRIzol reagent (Invitrogen) according to the manufacturer’s instructions. Total RNA was DNase-I treated (Invitrogen) and first strand synthesis was performed using SuperScript III reverse transcriptase (Invitrogen). cDNA was diluted 1:40 into ultrapure water, and mRNA expression determined using TaqMan assays (Invitrogen). For Sr-a determinations, primers were used as previously described (46). Relative expression was calculated using the ^ΔΔ^Ct method, as previously described (32, 47).

### Western blotting

Cellular lysates were prepared, and Western blotting and quantification were performed as previously described (32). All antibodies were from Cell Signaling Technologies.

### Flow cytometry

Cells were washed in PBS and removed from the wells via gentle pipetting into a 96-well 0.2 ml plate. Cells were then washed in PBS containing 1% BSA and pelleted at 500 × *g*. Cells were then incubated with 0.5 μg Fc block (CD16/32; BioLegend) for 30 min followed by incubation with the conjugated primary antibodies APC-CD36 (1:100; Miltenyl Biotec), FITC-SR-A (1:20; Miltenyl Biotec), and PE-SR-BI (1:20; Novus) for a further 30 min in a volume of 50 μl covered from light. Cells were then washed twice more with PBS containing 1% BSA and resuspended in a final volume of 200 μl. Cells were analyzed using a CyAn^TM^ ADP analyzer and fluorescence intensity was calculated as fold increase over unstained genotype and treatment controls by FlowJo software (Tree Star).

### In vivo RCT

In vivo RCT was performed as previously described (11, 48). In brief, BMDMs from Ampk β1^−/−^ and WT control mice were plated into 10 cm tissue culture plates. acLDL (50 μg/ml; BTI) pre-equilibrated with 5 μCi/ml [^3^H]cholesterol (Perkin Elmer) in DMEM supplemented with 5% LPDS (BTI) for 12 h, was then added to the BMDMs for a further 30 h. Cells were washed and harvested by gentle scraping. Approximately 8 × 10^6^ cells were injected into the intraperitoneal cavity of WT C57BL/6 mice. The radioactivity of each genotype preparation was determined prior to injection to account for changes in specific activity between isolations. Cellular cholesterol mass was also determined from each genotype to ensure equal lipid loading. Blood was collected at 48 h via cardiac puncture and livers were removed and weighed. Feces were collected over the 48 h period and total feces radioactivity was measured. Radioactivity was determined in serum, liver, and total feces (of equivalent wet weight) by LSC. All [^3^H]tracer measurements are expressed relative to the initial injected dose.

### Statistics

All results are shown as mean ± SEM. Results were analyzed using a two-tailed Student’s *t*-test or two-way ANOVA where appropriate using GraphPad Prism software. A Bonferonni post hoc test was used to test for significant differences revealed by the ANOVA. Significance was accepted at *P* ≤ 0.05.

## RESULTS

**Fig. 1.**
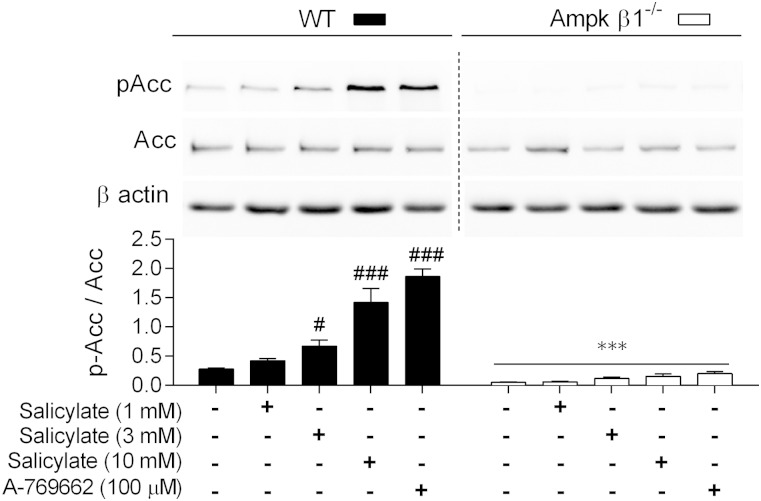
Activation of Ampk signaling in primary BMDM. The phosphorylation of Acc at Ser 79 was assessed as a measure of Ampk activation in response to increasing concentrations of salicylate as well as A-769662 in BMDM from WT and Ampk β1^−/−^ mice. Duplicate gels were run for total Acc determination. WT and Ampk β1^−/−^ samples are loaded on the same gel, dotted line represents a cropped lane. Gels are representative of three separate bone marrow isolations per genotype, performed in duplicate. Data represent mean ± SEM, where ***P < 0.001 compared within genotype and ^#^*P <* 0.05 and ^###^*P <* 0.001 are differences between treatment groups compared with vehicle control.

**Fig. 2.**
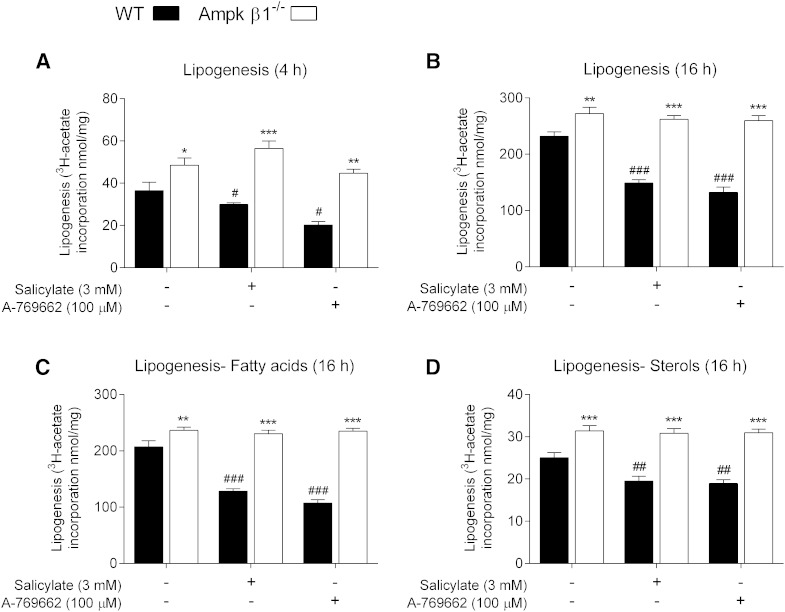
Activation of Ampk alters basal lipid synthesis and content. The incorporation of [^3^H]acetate into the total lipid fraction was measured as an indicator of lipogenesis after 4 h (A) and 16 h (B) treatments in the presence or absence of Ampk activators, A-769662 (100 μM), or salicylate (3 mM). Total lipids were then saponified and radioactivity determined from saponifiable (FAs) (C) and nonsaponifiable (sterols) (D) fractions, after 16 h. Data represent mean ± SEM and are from three separate bone marrow isolations per genotype, performed in triplicate, where *P < 0.05, **P < 0.01, and ***P < 0.001 are differences compared within genotype and ^#^*P <* 0.05, ^##^*P <* 0.01, and ^###^*P <* 0.001 are differences between treatment groups compared with vehicle control.

We first sought to determine the efficacy of the direct Ampk β1 activators in primary macrophages. Similar to hepatocytes (25), salicylate increased Ampk activity as indicated by increased phosphorylation of Acc1 (pSer 79) in a dose-dependent manner, as did A-769662, a result that we have shown previously (32) (**Fig. 1**). This effect was abolished in Ampk β1^−/−^ cells (Fig. 1). Neither treatment affected Ampk Thr172 phosphorylation (data not shown), which is in keeping with their allosteric mode of activation (24, 25, 27, 49). Phosphorylation of Acc1 by Ampk on Ser 79 inhibits its activity, resulting in reduced FA synthesis (22). Similar to primary hepatocytes (22, 39), deletion of the Ampk β1 subunit in BMDMs resulted in a higher lipogenic rate (incorporation of [^3^H]acetate into lipids) compared with WT controls (**Fig. 2A, B**). The dysregulation of lipid synthesis resulted in a higher incorporation of labeled acetate into both FAs and sterols in Ampk β1^−/−^ BMDMs compared with WT cells (Fig. 2C, D). Upon Ampk activation with the direct β1 activators salicylate and A-769662, both FA and sterol synthesis were inhibited in WT BMDMs, but were unaffected in the Ampk β1^−/−^ cells (Fig. 2A–D).

**Fig. 3.**
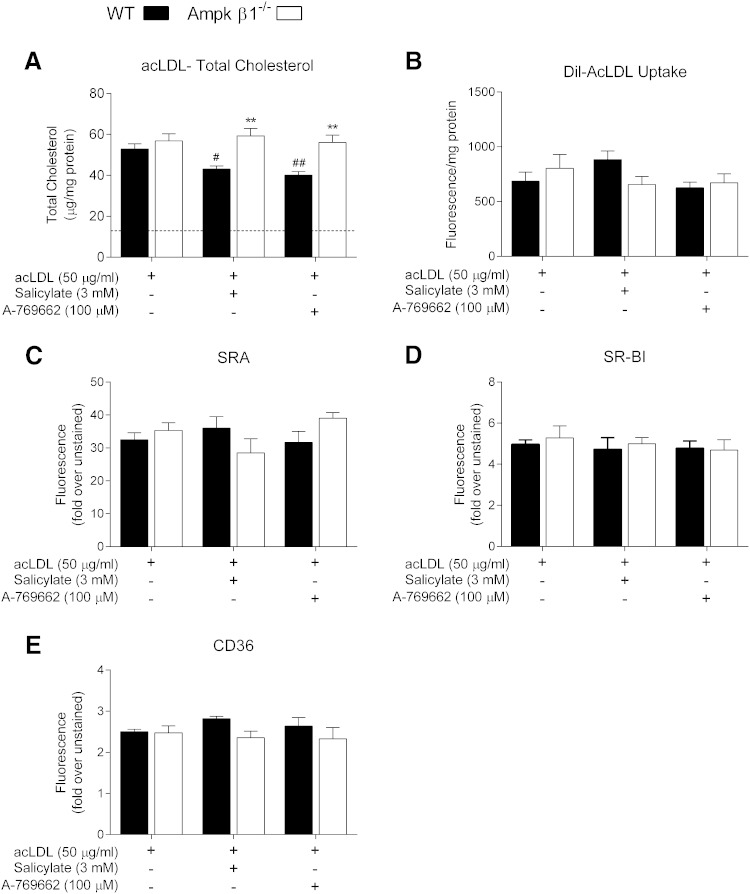
Activation of Ampk alters cholesterol uptake and foam cell formation. WT and Ampk β1^−/−^ BMDMs were lipid-loaded with acLDL (50 μg/ml) for 30 h in the presence or absence of the Ampk activators, salicylate (3 mM), or A-769662 (100 μM). After the loading period, total cholesterol (A) and the uptake of Dil-acLDL (B) (a further 2 h) were assessed. Surface expressions of scavenger receptors were determined by flow cytometry after lipid-loading in the presence or absence of Ampk activators. SR-A (C), SR-BI (D), and CD36 (E) were determined. Data represent mean ± SEM and are from three separate bone marrow isolations per genotype, performed in triplicate, where **P < 0.01 are differences compared within genotype and ^#^*P <* 0.05 and ^##^*P <* 0.01 are differences between treatment groups compared with vehicle (acLDL) control.

The progression of atherosclerosis stems from the unregulated uptake of modified lipoproteins by macrophages in the sub-endothelial space. We next treated WT and Ampk β1^−/−^ macrophages with acLDL to induce foam cell formation both in the presence and absence of Ampk activators. Exposure to acLDL caused a marked increase in cellular cholesterol content in cells from both genotypes (**Fig. 3A**), with no differences in triacylglyceride content (data not shown). When cells were lipid-loaded in the presence of salicylate or A-769662, there was a significant reduction in total cholesterol accumulation and foam cell formation in WT cells, but not Ampk β1^−/−^ cells. To further investigate how Ampk may regulate macrophage cholesterol metabolism, we monitored the uptake of Dil-acLDL in BMDM cells that had been lipid-loaded in the presence or absence of the Ampk β1 activators; however, there were no significant genotype or treatment differences (Fig. 3B). We next investigated the transcript and surface expression of the main scavenger receptor involved in acLDL uptake, SR-A, as well as SR-BI and CD36. There were no differences in transcript expression between genotype or treatment when cells were lipid-loaded in the presence of salicylate or A-769662 (supplementary Fig. 1A–C). To investigate further, we determined that the surface expression of SR-A, SR-BI, and CD36 were also unaltered (Fig. 3C–E). Because SR-A has been demonstrated to be the main effector by which acLDL is taken into the macrophage (50, 51), unaltered transcript and surface expression of SR-A (as well as other scavenger receptors) is in keeping with the notion that Ampk activation has no effect on acLDL uptake.

**Fig. 4.**
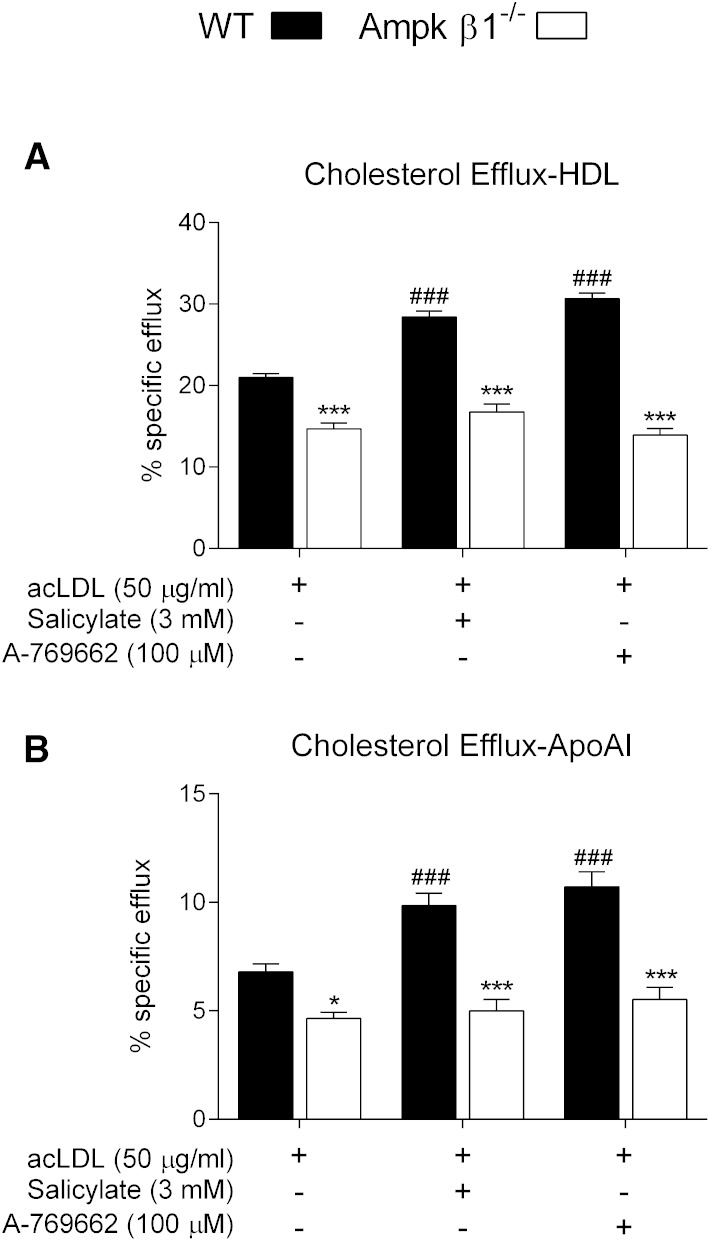
Activation of Ampk increases cholesterol efflux. BMDMs were prepared from WT and Ampk β1^−/−^ mice and cells were labeled with acLDL (50 μg/ml) supplemented with [^3^H]cholesterol (5 μCi/ml). Percent specific efflux was measured to HDL (A) and apoA-I (B). Data represent mean ± SEM and represent at least four separate bone marrow isolations per genotype, performed in triplicate, where *P < 0.05 and ***P < 0.001 are differences compared within genotype and ^###^*P <* 0.001 are differences between treatment groups compared with vehicle control.

The ability of macrophages to efflux cholesterol to extracellular acceptors is a critical component of RCT (9, 52). We treated BMDM cells with acLDL to induce foam cell formation, and then assessed cholesterol efflux to both HDL (mediated mainly by Abcg1) and lipid-poor apoA-I (mainly to Abca1) in the presence and absence of Ampk activators. Cholesterol efflux was lower in Ampk β1^−/−^ cells compared with WT control cells (**Fig. 4A, B**). Ampk activation via salicylate and A-769662 resulted in increased cholesterol efflux to both HDL and apoA-I (Fig. 4A, B), but only in WT macrophages, thus suggesting that Ampk-dependent processes contribute to macrophage RCT. Importantly, treatment with salicylate or A-769662 after the period of lipid loading did not alter cholesterol mass (supplementary Fig. 2), therefore specific activity was unaffected.

**Fig. 5.**
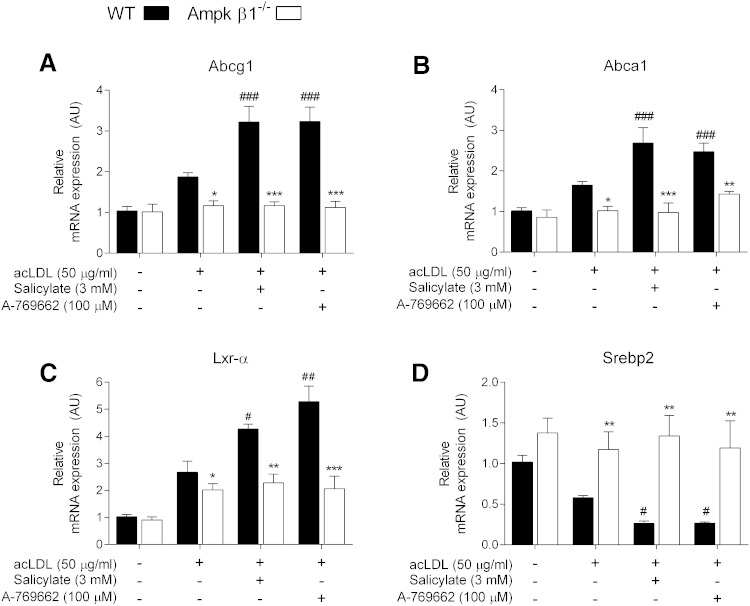
Ampk activation is associated with increased transporter expression. BMDMs were prepared from WT and Ampk β1^−/−^ mice and treated in the presence or absence of acLDL (50 μg/ml) for 30 h and then in the presence or absence of A-769662 (100 μM) and salicylate (3 mM) for a further 24 h. Transcript expression of the transporters Abcg1 (A) and Abca1 (B), as well as the transcriptional regulators Lxr-α (C) and Srebp2 (D) were determined. Transcripts are shown relative to WT control in the absence of acLDL and expressed relative to β actin. Data represent mean ± SEM and represent three separate bone marrow isolations per genotype, performed in quadruplicate, where *P < 0.05, **P < 0.01, and ***P < 0.001 are differences compared within genotype and ^#^*P* < 0.05, ^##^*P <* 0.01, and ^###^*P <* 0.001 are differences between treatment groups compared with vehicle (acLDL) control.

Given the significant effect of Ampk deletion on efflux to HDL and apoA-I, as well as the protective increase in efflux upon Ampk activation, we next assessed the transcript expression of the main cholesterol transporters in macrophages (48, 53). The expression of the Abcg1 and Abca1 transporters were reduced in Ampk β1^−/−^ BMDMs under vehicle-treated conditions (**Fig. 5A, B**), and both salicylate and A-769662 increased Abcg1 and Abca1 expression in WT but not Ampk β1^−/−^ cells (Fig. 5A, B). In addition to lipid transporter expression, we also monitored the expression of two important transcriptional regulators responsible for governing cholesterol homeostasis, liver X receptor α (Lxr-α) and sterol regulatory element binding protein (Srebp)2. Similar to the expression of Abca1 and Abcg1, Lxr-α expression was induced upon lipid loading, which occurred to a greater extent in WT BMDMs (Fig. 5C). Exogenous lipid loading suppressed Srebp2 expression, although this effect was prevented in the Ampk β1^−/−^ macrophages (Fig. 5D). Ampk activation was associated with further increases in Lxr-α (Fig. 5C) and further suppression of Srebp2 (Fig. 5D).

**Fig. 6.**
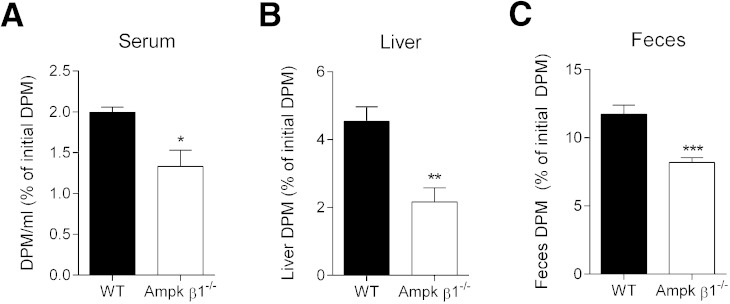
Ampk facilitates RCT in vivo. Macrophages were prepared from WT and Ampk β1^−/−^ mice and lipid-loaded with acLDL (50 μg/ml + [^3^H]cholesterol). WT and Ampk β1^−/−^ cells were then injected into the peritoneal cavity of WT recipients and, after 48 h, radioactivity was determined in the serum (A), liver (B), and total feces (C). Data represent mean ± SEM (n = 6 per group), where *P < 0.05, **P < 0.01, and ***P < 0.001.

We next investigated the role of macrophage Ampk in RCT in vivo. Radiolabeled, lipid-loaded macrophages from WT and Ampk β1^−/−^ mice (acLDL-[^3^H]cholesterol) were injected into WT mice. We assessed radioactivity in the serum, liver, and feces after 48 h (**Fig. 6A–C**). Consistent with the observed genotype difference in cholesterol efflux, radioactivity in the serum (*P <* 0.05), liver (*P <* 0.01), and feces (*P <* 0.001) were all significantly lower in mice that received macrophages from Ampk β1^−/−^ mice, compared with WT (Fig. 6). Taken together, these data are entirely consistent with a regulatory role for Ampk in mediating the reverse transport of cholesterol.

## DISCUSSION

Hyperlipidemia and other associated risk factors that predispose individuals to atherosclerosis lead to the over-abundance of modified LDL-cholesterol and unregulated uptake via scavenger receptors, ultimately causing foam cell formation and atherogenesis (7). We show that Ampk plays an integral role in regulating macrophage cholesterol accumulation. The deletion of the Ampk β1 subunit in macrophages results in a higher lipogenic rate and lower cholesterol efflux. Moreover, activation of Ampk using the direct Ampk β1 activators salicylate and A-769662 resulted in a protective decrease in lipogenesis, cholesterol accumulation, and foam cell formation, and an increase in cholesterol efflux.

Ampk inhibits multiple facets of lipid metabolism. The synthesis of FAs is inhibited via phosphorylation of Acc1 at Ser 79, which reduces the production of the FA precursor malonyl-CoA (22, 29). Ampk also phosphorylates Hmgcr at Ser 871 (in mice), the rate-limiting enzyme in cholesterol synthesis (31); however, the physiological role of this regulation has yet to be examined in detail. In addition, Ampk directly phosphorylates Srebp1c (Ser 372) and Srebp2 (site unknown) (54), which are master transcriptional regulators that govern lipid levels via modulation of FA and cholesterol synthetic pathways, respectively. Upon activation of Ampk in macrophages, the synthesis of FAs and sterols was inhibited in WT cells, but not Ampk β1^−/−^ cells (Fig. 2). Ampk β1 deficiency is associated with higher Srebp2 gene expression compared with WT control cells, and in response to Ampk activators, Srebp2 expression was significantly inhibited in WT cells, but not Ampk β1^−/−^ cells (Fig. 5D). The acute Ampk-specific inhibition of FA and sterol synthesis by salicylate and A-769662 after 4 h was likely mediated by the acute phosphorylation and inactivation of Acc1 and Hmgcr, respectively. However, during chronic experiments (24–30 h), the continued inhibition of lipogenesis and the modulation of lipid levels may reflect the net contribution of all affected pathways, including Srebp2. The relative contributions of the possible downstream regulators remain unclear.

In spite of differences in foam cell formation in the presence of Ampk activators (Fig. 3A), neither endogenous Ampk signaling, nor Ampk activation had any effect on the uptake of acLDL into lipid-loaded macrophages (Fig. 3B). This is consistent with previous studies demonstrating reductions in cholesterol accumulation with oxidized LDL and 5-aminoimidazole-4-carboxyamide ribonucleoside treatment, although there were no measures of uptake reported (55). In our experiments (supplementary Fig. 1) and those of Li et al. (55), this effect on foam cell formation was independent of alterations in scavenger receptor gene expression. Our investigation determined that the surface expression of key scavenger receptors (SR-BI and CD36), as well as the main receptor responsible for acLDL uptake, SR-A, were unaffected by Ampk signaling (Fig. 3C–E). This data suggests that Ampk activation alters cholesterol accumulations via uptake-independent mechanisms.

The ability of macrophages to efflux and the ability of HDL and other acceptors to transport cholesterol for the purpose of RCT have garnered therapeutic interest (15, 52, 56, 57). Many studies have used various cholesterol mobilization and transport protein knockout models to assess their role in macrophage RCT. Li et al. (55) demonstrated that 5-aminoimidazole-4-carboxyamide ribonucleoside, an indirect Ampk activator, increased Abcg1-mediated efflux to HDL in oxidized LDL-loaded macrophages and endothelial cells (58), although the authors did not assess efflux to apoA-I, nor did they note a basal difference with transient transfection of a dominant negative Ampk. Human monocyte-derived macrophages incubated with the Ampk activators metformin and heme also demonstrate an increase in cholesterol efflux and stemming of foam cell formation (59). In the current study we found that exposure to direct Ampk activators increased Abcg1 and Abca1 expression and cholesterol efflux to both HDL and apoA-I (Figs. 4, 5). Basal levels of efflux and associated transporter expression were lower in Ampk β1^−/−^ macrophages, indicating that Ampk is important for controlling endogenous cholesterol efflux. In addition, we show for the first time that macrophage Ampk plays a critical role in mediating efficient in vivo RCT (Fig. 6). Interestingly, the process of in vivo RCT was diminished in Ampk β1^−/−^ macrophages (∼30%) to a similar extent as cells that lack either Abca1 or Abcg1 (48, 53). This strongly suggests that Ampk is linked to important regulatory pathways that govern this process.

Our current study highlights a protective role for macrophage Ampk in regulating cholesterol metabolism. These effects are mediated through reductions in cholesterol synthesis under normal conditions and reductions in uptake and enhanced cholesterol efflux/RCT under lipid-loaded conditions. Importantly, we have tested A-769662 and salicylate and show that they reduce macrophage lipid synthesis and enhance cholesterol efflux via an Ampk β1-dependent pathway. While A-769662 has poor bioavailability (24), following salsalate and high-dose aspirin ingestion, salicylate is present in circulation at concentrations similar to those used in our studies (1–3 mM) (60, 61). Because Ampk β1 is the predominant subunit in human macrophages (33), it is interesting to speculate that the activation of macrophage Ampk might mediate a portion of the beneficial effects of these drugs on CVD. Future studies testing this hypothesis in mouse models of atherosclerosis are warranted.

## Supplementary Material

Supplemental Data

## References

[1] Skålén K. Gustafsson M. Rydberg E. K. Hultén L. M. Wiklund O. Innerarity T. L. Borén J. 2002 Subendothelial retention of atherogenic lipoproteins in early atherosclerosis. Nature. 417: 750–754. 12066187 10.1038/nature00804

[2] Hansson G. K. Robertson A. K. Soderberg-Naucler C. 2006 Inflammation and atherosclerosis. Annu. Rev. Pathol. 1: 297–329. 18039117 10.1146/annurev.pathol.1.110304.100100

[3] Moore K. J. Sheedy F. J. Fisher E. A. 2013 Macrophages in atherosclerosis: a dynamic balance. Nat. Rev. Immunol. 13: 709–721. 23995626 10.1038/nri3520 PMC4357520

[4] Smith J. D. Trogan E. Ginsberg M. Grigaux C. Tian J. Miyata M. 1995 Decreased atherosclerosis in mice deficient in both macrophage colony-stimulating factor (op) and apolipoprotein E. Proc. Natl. Acad. Sci. USA. 92: 8264–8268. 7667279 10.1073/pnas.92.18.8264 PMC41137

[5] Hansson G. K. Libby P. Schonbeck U. Yan Z. Q. 2002 Innate and adaptive immunity in the pathogenesis of atherosclerosis. Circ. Res. 91: 281–291. 12193460 10.1161/01.res.0000029784.15893.10

[6] Moore K. J. Freeman M. W. 2006 Scavenger receptors in atherosclerosis: beyond lipid uptake. Arterioscler. Thromb. Vasc. Biol. 26: 1702–1711. 16728653 10.1161/01.ATV.0000229218.97976.43

[7] Maxfield F. R. Tabas I. 2005 Role of cholesterol and lipid organization in disease. Nature. 438: 612–621. 16319881 10.1038/nature04399

[8] Legein B. Temmerman L. Biessen E. A. Lutgens E. 2013 Inflammation and immune system interactions in atherosclerosis. Cell. Mol. Life Sci. 70: 3847–3869. 23430000 10.1007/s00018-013-1289-1 PMC11113412

[9] Rader D. J. Alexander E. T. Weibel G. L. Billheimer J. Rothblat G. H. 2009 The role of reverse cholesterol transport in animals and humans and relationship to atherosclerosis. J. Lipid Res. 50(Suppl): S189–S194. 19064999 10.1194/jlr.R800088-JLR200 PMC2674717

[10] Ghosh S. Zhao B. Bie J. Song J. 2010 Macrophage cholesteryl ester mobilization and atherosclerosis. Vascul. Pharmacol. 52: 1–10. 19878739 10.1016/j.vph.2009.10.002 PMC2823947

[11] Ouimet M. Franklin V. Mak E. Liao X. Tabas I. Marcel Y. L. 2011 Autophagy regulates cholesterol efflux from macrophage foam cells via lysosomal acid lipase. Cell Metab. 13: 655–667. 21641547 10.1016/j.cmet.2011.03.023 PMC3257518

[12] Gelissen I. C. Harris M. Rye K. A. Quinn C. Brown A. J. Kockx M. Cartland S. Packianathan M. Kritharides L. Jessup W. 2006 ABCA1 and ABCG1 synergize to mediate cholesterol export to apoA-I. Arterioscler. Thromb. Vasc. Biol. 26: 534–540. 16357317 10.1161/01.ATV.0000200082.58536.e1

[13] Kennedy M. A. Barrera G. C. Nakamura K. Baldan A. Tarr P. Fishbein M. C. Frank J. Francone O. L. Edwards P. A. 2005 ABCG1 has a critical role in mediating cholesterol efflux to HDL and preventing cellular lipid accumulation. Cell Metab. 1: 121–131. 16054053 10.1016/j.cmet.2005.01.002

[14] Out R. Jessup W. Le Goff W. Hoekstra M. Gelissen I. C. Zhao Y. Kritharides L. Chimini G. Kuiper J. Chapman M. J. 2008 Coexistence of foam cells and hypocholesterolemia in mice lacking the ABC transporters A1 and G1. Circ. Res. 102: 113–120. 17967783 10.1161/CIRCRESAHA.107.161711

[15] Larach D. B. deGoma E. M. Rader D. J. 2012 Targeting high density lipoproteins in the prevention of cardiovascular disease? Curr. Cardiol. Rep. 14: 684–691. 22991041 10.1007/s11886-012-0317-3 PMC3517174

[16] Schwartz G. G. Olsson A. G. Ballantyne C. M. Barter P. J. Holme I. M. Kallend D. Leiter L. A. Leitersdorf E. McMurray J. J. Shah P. K. 2009 Rationale and design of the dal-OUTCOMES trial: efficacy and safety of dalcetrapib in patients with recent acute coronary syndrome. Am. Heart J. 158: 896–901. 19958854 10.1016/j.ahj.2009.09.017

[17] Boden W. E. Probstfield J. L. Anderson T. Chaitman B. R. Desvignes-Nickens P. Koprowicz K. McBride R. Teo K. Weintraub W.; AIM-HIGH Investigators. 2011 Niacin in patients with low HDL cholesterol levels receiving intensive statin therapy. N. Engl. J. Med. 365: 2255–2267. 22085343 10.1056/NEJMoa1107579

[18] Steinberg G. R. Kemp B. E. 2009 AMPK in health and disease. Physiol. Rev. 89: 1025–1078. 19584320 10.1152/physrev.00011.2008

[19] Hardie D. G. Ross F. A. Hawley S. A. 2012 AMPK: a nutrient and energy sensor that maintains energy homeostasis. Nat. Rev. Mol. Cell Biol. 13: 251–262. 22436748 10.1038/nrm3311 PMC5726489

[20] Iseli T. J. Walter M. van Denderen B. J. Katsis F. Witters L. A. Kemp B. E. Michell B. J. Stapleton D. 2005 AMP-activated protein kinase beta subunit tethers alpha and gamma subunits via its C-terminal sequence (186-270). J. Biol. Chem. 280: 13395–13400. 15695819 10.1074/jbc.M412993200

[21] Townley R. Shapiro L. 2007 Crystal structures of the adenylate sensor from fission yeast AMP-activated protein kinase. Science. 315: 1726–1729. 17289942 10.1126/science.1137503

[22] Fullerton M. D. Galic S. Marcinko K. Sikkema S. Pulinilkunnil T. Chen Z. P. O’Neill H. M. Ford R. J. Palanivel R. O’Brien M. 2013 Single phosphorylation sites in Acc1 and Acc2 regulate lipid homeostasis and the insulin-sensitizing effects of metformin. Nat. Med. 19: 1649–1654. 24185692 10.1038/nm.3372 PMC4965268

[23] Zhou G. Myers R. Li Y. Chen Y. Shen X. Fenyk-Melody J. Wu M. Ventre J. Doebber T. Fujii N. 2001 Role of AMP-activated protein kinase in mechanism of metformin action. J. Clin. Invest. 108: 1167–1174. 11602624 10.1172/JCI13505 PMC209533

[24] Cool B. Zinker B. Chiou W. Kifle L. Cao N. Perham M. Dickinson R. Adler A. Gagne G. Iyengar R. 2006 Identification and characterization of a small molecule AMPK activator that treats key components of type 2 diabetes and the metabolic syndrome. Cell Metab. 3: 403–416. 16753576 10.1016/j.cmet.2006.05.005

[25] Hawley S. A. Fullerton M. D. Ross F. A. Schertzer J. D. Chevtzoff C. Walker K. J. Peggie M. W. Zibrova D. Green K. A. Mustard K. J. 2012 The ancient drug salicylate directly activates AMP-activated protein kinase. Science. 336: 918–922. 22517326 10.1126/science.1215327 PMC3399766

[26] Göransson O. McBride A. Hawley S. A. Ross F. A. Shpiro N. Foretz M. Viollet B. Hardie D. G. Sakamoto K. 2007 Mechanism of action of A-769662, a valuable tool for activation of AMP-activated protein kinase. J. Biol. Chem. 282: 32549–32560. 17855357 10.1074/jbc.M706536200 PMC2156105

[27] Scott J. W. Ling N. Issa S. M. Dite T. A. O’Brien M. T. Chen Z. P. Galic S. Langendorf C. G. Steinberg G. R. Kemp B. E. 2014 Small molecule drug A-769662 and AMP synergistically activate naive AMPK independent of upstream kinase signaling. Chem. Biol. 21: 619–627. 24746562 10.1016/j.chembiol.2014.03.006

[28] Carlson C. A. Kim K. H. 1973 Regulation of hepatic acetyl coenzyme A carboxylase by phosphorylation and dephosphorylation. J. Biol. Chem. 248: 378–380. 4692841

[29] Munday M. R. Campbell D. G. Carling D. Hardie D. G. 1988 Identification by amino acid sequencing of three major regulatory phosphorylation sites on rat acetyl-CoA carboxylase. Eur. J. Biochem. 175: 331–338. 2900138 10.1111/j.1432-1033.1988.tb14201.x

[30] Beg Z. H. Allmann D. W. Gibson D. M. 1973 Modulation of 3-hydroxy-3-methylglutaryl coenzyme A reductase activity with cAMP and wth protein fractions of rat liver cytosol. Biochem. Biophys. Res. Commun. 54: 1362–1369. 4356818 10.1016/0006-291x(73)91137-6

[31] Clarke P. R. Hardie D. G. 1990 Regulation of HMG-CoA reductase: identification of the site phosphorylated by the AMP-activated protein kinase in vitro and in intact rat liver. EMBO J. 9: 2439–2446. 2369897 10.1002/j.1460-2075.1990.tb07420.x PMC552270

[32] Galic S. Fullerton M. D. Schertzer J. D. Sikkema S. Marcinko K. Walkley C. R. Izon D. Honeyman J. Chen Z. P. van Denderen B. J. 2011 Hematopoietic AMPK beta1 reduces mouse adipose tissue macrophage inflammation and insulin resistance in obesity. J. Clin. Invest. 121: 4903–4915. 22080866 10.1172/JCI58577 PMC3226000

[33] Dong C. Zhao G. Zhong M. Yue Y. Wu L. Xiong S. 2013 RNA sequencing and transcriptomal analysis of human monocyte to macrophage differentiation. Gene. 519: 279–287. 23458880 10.1016/j.gene.2013.02.015 PMC3666862

[34] Vane J. R. 1971 Inhibition of prostaglandin synthesis as a mechanism of action for aspirin-like drugs. Nat. New Biol. 231: 232–235. 5284360 10.1038/newbio231232a0

[35] Vane J. R. Bakhle Y. S. Botting R. M. 1998 Cyclooxygenases 1 and 2. Annu. Rev. Pharmacol. Toxicol. 38: 97–120. 9597150 10.1146/annurev.pharmtox.38.1.97

[36] Khan Q. Mehta J. L. 2005 Relevance of platelet-independent effects of aspirin to its salutary effect in atherosclerosis-related events. J. Atheroscler. Thromb. 12: 185–190. 16141621 10.5551/jat.12.185

[37] Hennekens C. H. Dyken M. L. Fuster V. 1997 Aspirin as a therapeutic agent in cardiovascular disease: a statement for healthcare professionals from the American Heart Association. Circulation. 96: 2751–2753. 9355934 10.1161/01.cir.96.8.2751

[38] Higgs G. A. Salmon J. A. Henderson B. Vane J. R. 1987 Pharmacokinetics of aspirin and salicylate in relation to inhibition of arachidonate cyclooxygenase and antiinflammatory activity. Proc. Natl. Acad. Sci. USA. 84: 1417–1420. 3103135 10.1073/pnas.84.5.1417 PMC304441

[39] Dzamko N. van Denderen B. J. Hevener A. L. Jorgensen S. B. Honeyman J. Galic S. Chen Z. P. Watt M. J. Campbell D. J. Steinberg G. R. 2010 AMPK beta1 deletion reduces appetite, preventing obesity and hepatic insulin resistance. J. Biol. Chem. 285: 115–122. 19892703 10.1074/jbc.M109.056762 PMC2804155

[40] Quinn J. M. Tam S. Sims N. A. Saleh H. McGregor N. E. Poulton I. J. Scott J. W. Gillespie M. T. Kemp B. E. van Denderen B. J. 2010 Germline deletion of AMP-activated protein kinase beta subunits reduces bone mass without altering osteoclast differentiation or function. FASEB J. 24: 275–285. 19723702 10.1096/fj.09-137158 PMC2797037

[41] Dong F. Mo Z. Eid W. Courtney K. C. Zha X. 2014 Akt inhibition promotes ABCA1-mediated cholesterol efflux to ApoA-I through suppressing mTORC1. PLoS ONE. 9: e113789. 25415591 10.1371/journal.pone.0113789 PMC4240609

[42] Kushiyama A. Sakoda H. Oue N. Okubo M. Nakatsu Y. Ono H. Fukushima T. Kamata H. Nishimura F. Kikuchi T. 2013 Resistin-like molecule beta is abundantly expressed in foam cells and is involved in atherosclerosis development. Arterioscler. Thromb. Vasc. Biol. 33: 1986–1993. 23702657 10.1161/ATVBAHA.113.301546

[43] Han X. Kitamoto S. Lian Q. Boisvert W. A. 2009 Interleukin-10 facilitates both cholesterol uptake and efflux in macrophages. J. Biol. Chem. 284: 32950–32958. 19776020 10.1074/jbc.M109.040899 PMC2781710

[44] Bligh E. G. Dyer W. J. 1959 A rapid method of total lipid extraction and purification. Can. J. Biochem. Physiol. 37: 911–917. 13671378 10.1139/o59-099

[45] Fullerton M. D. Hakimuddin F. Bakovic M. 2007 Develop­mental and metabolic effects of disruption of the mouse CTP:phosphoethanolamine cytidylyltransferase gene (Pcyt2). Mol. Cell. Biol. 27: 3327–3336. 17325045 10.1128/MCB.01527-06 PMC1899976

[46] DeWitte-Orr S. J. Collins S. E. Bauer C. M. Bowdish D. M. Mossman K. L. 2010 An accessory to the ‘Trinity’: SR-As are essential pathogen sensors of extracellular dsRNA, mediating entry and leading to subsequent type I IFN responses. PLoS Pathog. 6: e1000829. 20360967 10.1371/journal.ppat.1000829 PMC2847946

[47] Livak K. J. Schmittgen T. D. 2001 Analysis of relative gene expression data using real-time quantitative PCR and the 2(-Delta Delta C(T)) method. Methods. 25: 402–408. 11846609 10.1006/meth.2001.1262

[48] Wang X. Collins H. L. Ranalletta M. Fuki I. V. Billheimer J. T. Rothblat G. H. Tall A. R. Rader D. J. 2007 Macrophage ABCA1 and ABCG1, but not SR-BI, promote macrophage reverse cholesterol transport in vivo. J. Clin. Invest. 117: 2216–2224. 17657311 10.1172/JCI32057 PMC1924499

[49] Scott J. W. van Denderen B. J. Jorgensen S. B. Honeyman J. E. Steinberg G. R. Oakhill J. S. Iseli T. J. Koay A. Gooley P. R. Stapleton D. 2008 Thienopyridone drugs are selective activators of AMP-activated protein kinase beta1-containing complexes. Chem. Biol. 15: 1220–1230. 19022182 10.1016/j.chembiol.2008.10.005

[50] Ling W. Lougheed M. Suzuki H. Buchan A. Kodama T. Steinbrecher U. P. 1997 Oxidized or acetylated low density lipoproteins are rapidly cleared by the liver in mice with disruption of the scavenger receptor class A type I/II gene. J. Clin. Invest. 100: 244–252. 9218499 10.1172/JCI119528 PMC508185

[51] Suzuki H. Kurihara Y. Takeya M. Kamada N. Kataoka M. Jishage K. Ueda O. Sakaguchi H. Higashi T. Suzuki T. 1997 A role for macrophage scavenger receptors in atherosclerosis and susceptibility to infection. Nature. 386: 292–296. 9069289 10.1038/386292a0

[52] Khera A. V. Cuchel M. de la Llera-Moya M. Rodrigues A. Burke M. F. Jafri K. French B. C. Phillips J. A. Mucksavage M. L. Wilensky R. L. 2011 Cholesterol efflux capacity, high-density lipoprotein function, and atherosclerosis. N. Engl. J. Med. 364: 127–135. 21226578 10.1056/NEJMoa1001689 PMC3030449

[53] Wang M. D. Franklin V. Marcel Y. L. 2007 In vivo reverse cholesterol transport from macrophages lacking ABCA1 expression is impaired. Arterioscler. Thromb. Vasc. Biol. 27: 1837–1842. 17541020 10.1161/ATVBAHA.107.146068

[54] Li Y. Xu S. Mihaylova M. M. Zheng B. Hou X. Jiang B. Park O. Luo Z. Lefai E. Shyy J. Y. 2011 AMPK phosphorylates and inhibits SREBP activity to attenuate hepatic steatosis and atherosclerosis in diet-induced insulin-resistant mice. Cell Metab. 13: 376–388. 21459323 10.1016/j.cmet.2011.03.009 PMC3086578

[55] Li D. Wang D. Wang Y. Ling W. Feng X. Xia M. 2010 Adenosine monophosphate activated protein kinase induces cholesterol efflux from macrophage-derived foam cells and alleviates atherosclerosis in apolipoprotein E-deficient mice. J. Biol. Chem. 285: 33499–33509. 20713354 10.1074/jbc.M110.159772 PMC2963409

[56] Khera A. V. Rader D. J. 2013 Cholesterol efflux capacity: full steam ahead or a bump in the road? Arterioscler. Thromb. Vasc. Biol. 33: 1449–1451. 23766382 10.1161/ATVBAHA.113.301519

[57] Rader D. J. Tall A. R. 2012 The not-so-simple HDL story: is it time to revise the HDL cholesterol hypothesis? Nat. Med. 18: 1344–1346. 22961164 10.1038/nm.2937

[58] Li D. Zhang Y. Ma J. Ling W. Xia M. 2010 Adenosine monophosphate activated protein kinase regulates ABCG1-mediated oxysterol efflux from endothelial cells and protects against hypercholesterolemia-induced endothelial dysfunction. Arterioscler. Thromb. Vasc. Biol. 30: 1354–1362. 20395595 10.1161/ATVBAHA.110.204230

[59] Wan X. Huo Y. Johns M. Piper E. Mason J. C. Carling D. Haskard D. O. Boyle J. J. 2013 5′-AMP-activated protein kinase-activating transcription factor 1 cascade modulates human monocyte-derived macrophages to atheroprotective functions in response to heme or metformin. Arterioscler. Thromb. Vasc. Biol. 33: 2470–2480. 24051143 10.1161/ATVBAHA.113.300986

[60] Day R. O. Graham G. G. Bieri D. Brown M. Cairns D. Harris G. Hounsell J. Platt-Hepworth S. Reeve R. Sambrook P. N. 1989 Concentration-response relationships for salicylate-induced ototoxicity in normal volunteers. Br. J. Clin. Pharmacol. 28: 695–702. 2611090 10.1111/j.1365-2125.1989.tb03562.x PMC1380040

[61] Hundal R. S. Petersen K. F. Mayerson A. B. Randhawa P. S. Inzucchi S. Shoelson S. E. Shulman G. I. 2002 Mechanism by which high-dose aspirin improves glucose metabolism in type 2 diabetes. J. Clin. Invest. 109: 1321–1326. 12021247 10.1172/JCI14955 PMC150979

