# Supplementary material for: Salicylate improves macrophage cholesterol homeostasis via activation of Ampk
Source: J Lipid Res. 2015 May;56(5):1025–33. doi: 10.1194/jlr.M058875 (PMC4409279; doi:10.1194/jlr.M058875)
Supplement: Supplemental Data [file supp_56_5_1025__index.html]

Salicylate improves macrophage cholesterol homeostasis via activation of Ampk — Salicylate improves macrophage cholesterol homeostasis via activation of Ampk — Supplemental Data 

# Salicylate improves macrophage cholesterol homeostasis via activation of Ampk

## Supplemental Data

**Files in this Data Supplement:**

- Supplemental Figures - Supplemental Figures
