# Supplementary material for: Salicylate improves macrophage cholesterol homeostasis via activation of Ampk
Source: J Lipid Res. 2015 May;56(5):1025–33. doi: 10.1194/jlr.M058875 (PMC4409279; doi:10.1194/jlr.M058875)

Supplemental Figure Legend:

Supplemental Figure S1. Scavenger receptor expression in unaltered. Transcript expression of (a) the scavenger receptor-A (SR-A), (b) SR-BI and (c) CD36 were determined ± acLDL (50 µg/ml) and ± A-769662 (100 µM) and salicylate (3 mM). Transcripts are shown relative to WT control in the absence of acLDL and expressed relative to β actin. Data represent mean ± SEM, and are from 3 separate bone marrow isolations per genotype, performed in triplicate, where $^{\#}p<0.05$ are differences between treatment groups compared to no acLDL control.

Supplemental Figure S2. Total cholesterol content is unaltered prior to efflux. WT and Ampk $\beta1^{-/-}$ BMDM were lipid-loaded with acLDL (50 µg/ml) for 30 h. Lipid containing media was then removed and cells were equilibrated in media containing 0.2% BSA in the presence or absence of the Ampk activators, salicylate (3 mM) or A-769662 (100 µM) for a further 16 hours. Total cholesterol was then assessed. Data represent mean ± SEM, and are from 3 separate bone marrow isolations per genotype, performed in quadruplicate.

Supplemental Figure S1

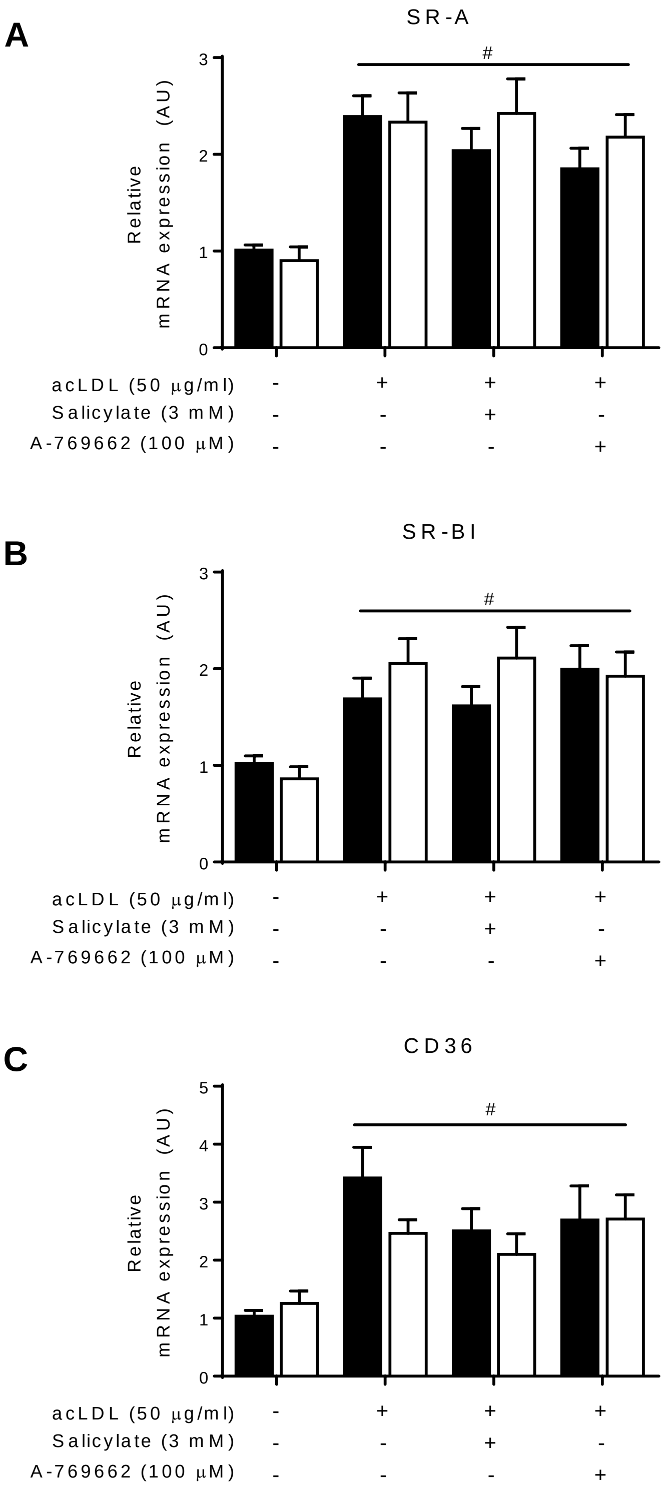

Supplemental Figure S2

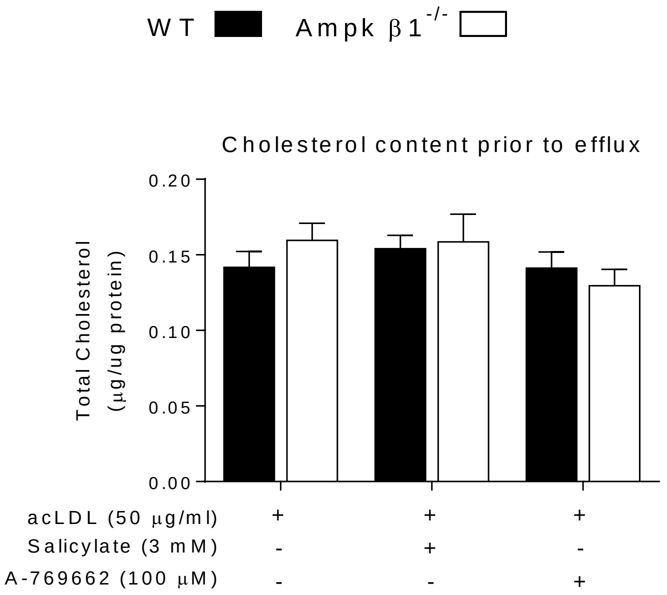

Supplement: Supplemental Data [file supp_M058875_jlr.M058875-1.pdf]
